# Alcohol and Happiness

**Published:** 1896-03

**Authors:** Justus Gaule


### Alcohol and Happiness.

The body uses its powers in resisting the outside forces which act upon it. Normally, there is a balance between body and environment. If environment prevails we are discouraged; if we are able to prevail, our spirits rise and our happiness grows. And it is not for the moment only, but we compare the accumulated impression of the powers outside of us with the powers which our brains develop, and are happy or unhappy according as we feel our superiority or otherwise. Just how much does alcohol interfere in this balance of power? It clearly can not lessen the power of outside influences which harm us; it can as clearly not increase our own powers in so far as they enter into this conflict with the outside world—it rather makes us less skillful and able. What can it do, then? It can deceive us. It dulls our appreciation of powers outside of us until they seem so much smaller that we are sure we can conquer them, and so we gain a feeling of satisfaction. Nine-tenths of those who take strong drink seek this feeling in alcohol. This is their "refreshing" at eventide, their "rest from the day's cares," their forgetfulness of sorrows; but it rests upon a deceit, and at the least trial falls into ruin. He who to-day forgets is not any stronger to-morrow, and so is constantly tempted to a new appeal to his false friend until his senses are so dulled that every duty is forgotten. His holiest interests are but shadows and mist before his eyes, and he knows nothing more but thirst for the deceitful drink. Even the defenders of alcohol at last call a halt; but they have forgotten that the first steps are much more easily undone than the later ones, when the brain has in a measure lost its power to control. They do not forget through malice, but because they have not rightly understood the physiological effect of alcohol.—Dr. Justus Gaule *in Popular Science Monthly.*

It is said that the tendons found in the tail of a dog make better sutures than either catgut or kangaroo tendon, when properly prepared in sublimate.

